# Involvement of Bacterial Extracellular Membrane Nanovesicles in Infectious Diseases and Their Application in Medicine

**DOI:** 10.3390/pharmaceutics14122597

**Published:** 2022-11-25

**Authors:** Konstantin A. Lusta, Anastasia V. Poznyak, Larisa Litvinova, Paolo Poggio, Alexander N. Orekhov, Alexandra A. Melnichenko

**Affiliations:** 1 Institute for Atherosclerosis Research, Osennyaya Street 4-1-207, 121609 Moscow, Russia; 2 Center for Immunology and Cellular Biotechnology, Immanuel Kant Baltic Federal University, 6 Gaidara Street, 236001 Kaliningrad, Russia; 3 Unit for Study of Aortic, Valvular and Coronary Pathologies, Centro Cardiologico Monzino IRCCS, 20137 Milan, Italy; 4 Institute of General Pathology and Pathophysiology, 8 Baltiiskaya Street, 125315 Moscow, Russia

**Keywords:** exosomes, bacterial membrane, vesicles, infection, infectious disease

## Abstract

Bacterial extracellular membrane nanovesicles (EMNs) are attracting the attention of scientists more and more every year. These formations are involved in the pathogenesis of numerous diseases, among which, of course, the leading role is occupied by infectious diseases, the causative agents of which are a range of Gram-positive and Gram-negative bacteria. A separate field for the study of the role of EMN is cancer. Extracellular membrane nanovesicles nowadays have a practical application as vaccine carriers for immunization against many infectious diseases. At present, the most essential point is their role in stimulating immune response to bacterial infections and tumor cells. The possibility of nanovesicles’ practical use in several disease treatments is being evaluated. In our review, we listed diseases, focusing on their multitude and diversity, for which EMNs are essential, and also considered in detail the possibilities of using EMNs in the therapy and prevention of various pathologies.

## 1. Introduction

The development of new technologies, such as atomic force microscopy, cryogenic electron microscopy, immunoelectron microscopy, confocal laser scanning microscopy, laser correlation spectroscopy, dynamic light scattering, high-resolution mass spectrometry, genetic and protein engineering made it possible to identify and study the basic structure and function of extracellular membrane nanovesicles (EMN) secreted by almost all types of eukaryotic and prokaryotic cells [1]. Bacterial and eukaryotic EMN are practically indistinguishable in size but differ in their constituent components. However, due to the lack of sufficient knowledge, we cannot yet accurately mark these two different types of vesicles in mammalian organisms’ body fluids. Various authors have proposed many different terms for these nanostructures, such as exosomes, exovesicles, exosome-like vesicles, ectosomes, cellular vesicles, extracellular vesicles, microvesicles, microparticles, endosomes, oncosomes, apoptotic bodies, and many others (predominantly for eukaryotic EMN) [2,3], and outer membrane vesicles, extracellular membrane nanovesicles, shedding microvesicles, blebbing vesicles, nanovesicles, or nanoparticles (predominantly for bacterial EMN) [4,5,6,7,8,9].

**Figure 1:**
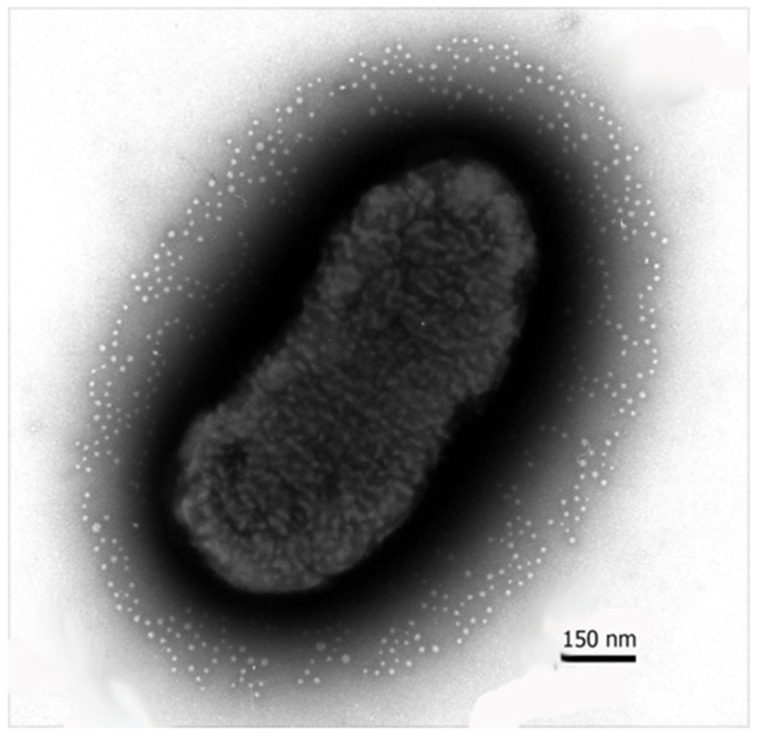
Electron micrograph of negatively contrasted bacterium *Aeromons hydrophila* H 1-6-05 (ammonium molybdate stain). Accumulations of numerous extracellular membrane nanovesicles on the surface, outside of bacterial cell, is presented. The spherical EMN are 20 to 50 nm in diameter. (Material is provided by Konstantin A. Lusta).

It has been established that all types of prokaryotes, including Gram-positive and Gram-negative bacteria, mycobacteria, and Archaea, secrete EMNs in the environment [5,9,10,11,12]. EMN represents spherical structures surrounded by a bilayer membrane formed from the mother cell membrane. The size of majority of EMN is 20–300 nm in diameter [5,13] (Figure 1), and they cannot replicate by ourselves [14] but are capable to transfer a variety of compounds from their parent cells [15].

EMN constitutes a significant fraction of bacterial cellular material, as was measured by several different methods [16]. Their content mainly reflects the composition of bacterial membranes, as well as components of the periplasm and cytoplasm, including proteins, lipopolysaccharides, phospholipids, peptidoglycan, RNA, and DNA molecules [12,17,18]. They also contain various biologically active proteins, such as invasins, transporters, receptors, signaling molecules, virulence factors, hydrolytic enzymes, and many others [19,20]. After the detailed proteomic analysis of EMN isolated from various bacteria, it was found that they contain thousands of different proteins [20,21]. Among them were revealed membrane-associated proteins (36%), periplasmic proteins (16%), and cytosolic proteins (32%) [22]. The proteins concerned with EMN building-up, disposal of harmful substances, fighting against competing organisms, targeted EMN delivery, DNA and RNA transfer, and alteration in host immunity were identified [23]. EMN proteins were revealed, such as AcfD lipoprotein, TolC receptor, ferrichrome protein, phospholipase A1, β-glucosidase, TSX channel-forming protein, LC porin, colicin I receptor, vitamin B12 receptor, chaperone protein HtpG, elongation factor Tu, ATP synthase α chain, 60-kDa chaperonin, AmpC β-lactamase, AbOmpA, chaperonin GroEL, cytolethal toxins and many others [24,25,26,27,28]. Apart from biologically active proteins, both DNA and RNA loaded into EMN can activate host immune responses [29].

EMN’s small size allows them to overcome biological barriers [30]. Exosomes participate in a wide variety of biological processes, both physiological and pathological [12]. EMN content composition is formed by the controlled selection of specific compounds [28]. The studies confirm the EMN’s non-random formation: enrichment in some components and exclusion of others indicates that extracellular vesicles are a product of biological mechanisms for bacterial adaptation to environmental conditions [31,32]. Nevertheless, the biochemical composition of EMN retains the characteristics of the mother cell [5,12,13,33,34]. Bacteria and tumor cells use extracellular vesicles as a transfer mechanism and antigen-presenting structures for distribution throughout the organism and protection from immune monitoring [35,36]. Pathogenic bacteria’s EMN causes infection to host cells as these vesicles can deliver virulence factors [37,38]. Moreover, extracellular vesicles found in all fluids of animals and humans are an information source about many processes and disturbances in them [12,39]. Since EMN exhibit the specific feature of immunogenic nanoparticles, they are nowadays employed in antibacterial immunotherapy, as well as to diagnosticate and medicate cancer [40]. An important EMN’s quality for this purpose is the expedient facility of intended transformation of their properties [41]. Following the virulence criteria, emitted by pathogenic bacteria, EMN represents a new type of infectious agent, which determines the need to correct approaches in the control of bacterial infections. Thuswise, understanding the essence of EMN is of paramount importance for the development of both diagnostic and therapeutic methods [15,42]. In this review, we summarized the data on the implication of EMN in various diseases, as well as their prevention and treatment options. This can be essential for determining the direction of future investigations.

## 2. EMN Functions

Currently, significant progress has been made in understanding the biological functions and mechanisms of EMN formation [5,43]. The study shows the extracellular vesicles both for their physiological and ecological function, as well as for bacterial pathogenicity [44]. EMN plays a variety of biological roles, including protein secretion, toxins delivery to host cells, and lysis of other bacteria [45,46]. EMN is an important intercellular communication mediator, making the exchange of substances and information between cells both in the microbial community and bacteria-eucaryotes interaction [6,45,47,48]. They perform microbe-host interactions [6,15,44], disposal of unnecessary compounds, storage of useful substances [5,17], immune activation or suppression, attachment to various cells, and internalization [43,49]. EMN gives input to the survival and protection of bacteria, is produced in response to stress, and is a tool for bacterial virulence [50,51,52,53].

Extracellular secretion of various substances using EMN is the main mechanism by which pathogenic bacteria administer the intoxication of host cells [38]. These virulence factors alter cell biology and allow parental bacteria to inhabit the host [54]. Apart from the toxigenic effects, EMN’s virulence factors activate the host immune response [55].

EMN secretion system is employed by bacteria to modulate maternal bacterial defense and disrupt host cell function [56,57,58]. Pathogenic bacteria’s EMN contains a variety of virulence factors, such as toxins, adhesins, LPS, hydrolytic enzymes, immunomodulators, pathogen-associated molecular patterns (PAMPs) representing an extensive set of microbial substances with conserved motifs, linked to pathogen infection, serving as ligands for host pattern recognition molecules, including Toll-like receptors (TLRs). All of these components exert a toxigenic effect. EMN can be considered a type of bacterial offensive instrumentality [59].

EMN-packaged toxins have several advantages compared to freely secreted compounds:

(a) they deliver proteins without their enzymatic degradation and the contents dilution in the environment, directly into host cells [60]; (b) EMN can deliver virulence factors at high concentrations to distant targets [61]; (c) EMN contain adhesins on the surface, which help them to interact with host cell’s plasma membrane [62]; (d) EMN provide optimal conditions for the folding of toxin proteins [63]; (e) EMN are used to deliver virulence factors that affect only the intended target [45]. The toxin’s delivery by EMN is a powerful mechanism for the virulence of pathogenic bacteria that modulate immunity and cause various diseases.

## 3. Diseases Caused by Variety of Pathogen’s EMN

### 3.1. Diseases of the Upper and Lower Respiratory Tract

These diseases are caused by bacteria such as *Haemophilus influenzae*, *Pseudomonas aeruginosa*, *Streptococcus pneumoniae*, and *Moraxella catarrhalis* [64]. The main microbe-expressed virulence factors are CopB, a surface protein that participates in the extraction of iron from lactoferrin or transferrin [65], and OMPs: UspA1 and UspA2, associated with adhesion to the epithelium [66]. *M. catarrhalis* secrete EMN containing surface proteins UspA1 and UspA2, which mediate interaction with the C3 component of complement [67]. H. influenzae also produces EMN that contain P5 adhesin, IgA endopeptidase, a serine protease, DNA, and heme utilization protein, indicating their virulence [53]. Upon EMN internalization, they colocalize with caveolin and become involved in caveolae-mediated endocytosis [68]. When interacting with epithelial cells, EMN stimulates Toll-like receptors, and the release of cytokines IL-6, IL-8, and IL-12, and also the antimicrobial peptide cathelicidin LL-37 [53]. The chain of these events determines EMN’s dynamic role in *H. influenzae* pathogenesis [69]. The vesicles interact directly with B cell receptor leading to B cell activation [70] that resulted in a substantial initiating of CD69 and CD86 cell surface molecules and increased secretion of IgM and IgG by B cells that were incubated with *H. influenzae* EMN [71].

### 3.2. Pneumonia

Lung inflammation can be caused by variety of pathogenic bacteria, such as *Acinetobacter baumannii*, Moraxella catarrhalis, Aeromonas hydrophila, *Pasteurella multocida*, *Burkholderia cepacia*, *Haemophilus influenzae*, *Escherichia coli* (ExPEC), *Legionella pneumophila*, *Pseudomonas aeruginosa*, *Pasteurella pestis*, *Streptococcus pneumonia* (pneumococcus), and *S. pneumonia* [72]. These bacteria produce the protein toxin pneumolysin (Ply), a pore-forming hemolysin that is an essential component launching NO production in macrophages [73]. NO has both beneficial and detrimental effects on acute inflammation [74,75]. NO is produced from L-arginine by inducible NO synthase (iNOS) [76]. Pro-inflammatory cytokines, for example, TNF-α, IL-1, and IFN-γ can activate iNOS [77]. The pathway regulation is mediated by interferon regulatory factor 1 that binds to the iNOS promoter and activates iNOS transcription [78]. *S. pneumoniae* EMN involvement in the pathogenesis of pulmonary inflammation was confirmed in experiments with polymyxin B (LPS antagonist) which blocks EMN internalization into respiratory tract epithelium. These vesicles enclose, apart from Ply, such virulence factors as IgA protease, pyruvate oxidase SpxB, metalloprotease ZmpB, and adhesion proteins [79,80].

*Acinetobacter baumannii* also caused pneumonia due to the cytotoxic activity of OmpA and tissue-degrading enzymes packaged in EMN. These vesicles interact with lipid rafts and after internalization, EMN induces host cell apoptosis [81]. *Pseudomonas aeruginosa* possesses several EMN-associated virulence factors. A total of 338 proteins have been identified in nanovesicles, including alkaline protease and a transmembrane regulator (Cif), which impairs ion transport, osmotic pressure, membrane permeability, and protein synthesis [60]. To confirm EMN involvement, isolated vesicles were injected into the lungs of knockout mice, the consequence of which was inflammation and increased concentrations of chemokines and cytokines. Thus, *P. aeruginosa* EMN causes lung inflammation without live bacteria [82]. Bacterial EMN was exploited recently as a high-potential vaccine against pneumonia [83].

### 3.3. Tuberculosis

The infectious agent of this severely virulent disease is the bacterial pathogen *Mycobacterium tuberculosis* that caused over a billion deaths in the past 200 years [84]. *M. tuberculosis* throws off EMN containing immunologically competent substances capable to seize host cells [85]. M. tuberculosis EMN was revealed to contain the virulence factors, such as lipoproteins LpqH, LprA, LppX, and PstS1, which represent the TLR2 ligands impairing the antigen presentation in macrophages. When EMN was injected in mice, an enhance in granulomatous inflammation in the lungs was registered [86]. Moreover, M. tuberculosis EMN stimulated an increase in co-stimulatory molecules CD80, a higher expression of CD86, the forthcoming of many cytokines, like TNF-α and IL-6, and increased superoxide anion production in human macrophages. Thus, TLR2 ligation and superoxide anion production result in neutrophils and macrophages autophagy [87].

### 3.4. Cystic Fibrosis (CF)

This disease leads to chronic respiratory infections and pancreatic insufficiency. Pathogenic bacteria like *Staphylococcus aureus*, *Burkholderia cepacian*, *Stenotrophomonas maltophilia*, and *Pseudomonas aeruginosa* are involved in this disease [88]. CF is caused by the presence of mutations in the transmembrane regulatory protein (CFTR) required for the mucociliary system. EMN-packaged Cif toxin promotes CTFR degradation. Cif introduces into host cells after the EMN fusion with lipid rafts and triggers lysosomal degradation of CFTR [60,89,90].

### 3.5. Whooping Cough

Bacterium *Bordetella pertussis* causes this infection disease, which produces adenylate cyclase hemolysin (AC-Hly), pertussin toxin, agglutinogens, and adenylate cyclase toxin (ACT). These virulence factors are responsible for the immune response [91]. *B. pertussis* in the patient’s lungs excretes EMN containing ACT (EMN-ACT), indicating their involvement in pathogenesis. This toxin intoxicates cells as a result of intracellular cAMP production [92]. ACT exerts influence on cells in the presence of the CD11b/CD18 toxin receptor [93]. ACT intoxication is blocked by antitoxins and anti-CD11b antibodies but not by cytochalasin-D; however, EMH-ACT is not affected by antibodies but is blocked by cytochalasin-D. Thus, EMN can deliver ACT to host cells through alternative mechanisms [94].

### 3.6. Sepsis

This syndrome is characterized by a systemic inflammatory response and immune dysregulation [95]. In these circumstances activating the inflammatory cascade in endothelial cells occur, and the release of pro-inflammatory and anti-inflammatory mediators increases [96,97]. The bacteria, for example, *Streptococcus pyogenes*, *Pseudomonas aeruginosa*, *Escherichia coli*, *Pasteurella multocida*, and *Klebsiella* spp. are the causative agents. EMN secreted by these bacteria initiates an inflammatory response independently of the parent bacteria. The symptoms of sepsis were observed when EMN was administered to rats [96]. Factors causing sepsis are PAMPs such as LPS, muramyl dipeptide, lipoteichoic acid, and bacterial DNA. They are recognized by PRRs, such as C-type lectin receptors, TLRs, and NOD-like receptors. The association of PAMP and PRR triggers the signaling cascades. NF-κB transcription factors, and activator protein-1, regulate the cytokine expression, such as IL-6, IL-8, IL-12, and TNF-α [98].

### 3.7. Plague

This is a deadly disease triggered by the *Yersinia pestis*. [99]. The disease has three main forms: pneumonic, septicemic, and bubonic. Death can occur in less than 2 weeks. [100]. *Y. pestis* produces EMN that contains multiple virulence-associated proteins, including Ail adhesin and Pla protease [101]. Catalytically active Pla executes plasminogen activation and degradation of α2-antiplasmin [102]. Interaction of EMN, containing Pla, with substrates, such as plasminogen, determines the course of the infectious process [101].

### 3.8. Gonorrhea

The bacterium *Neisseria gonorrhoeae* is an infectious agent that causes this sexually transmitted disease. The surface components, such as OMP are involved in pathogenesis. In emitted by N. gonorrhoeae EMN, 168 proteins were identified. Among them, 34 were found both in cell membranes and in EMN. Exhaustion of the OMP homologue, LptD, has been shown to cause loss of viability in *N. gonorrhoeae*, such as in the OMP1985 deletion mutant. These results indicate that EMN contains virulence factors, which are important determinants that represent promising targets for new therapeutic approaches [103].

### 3.9. Peptic Ulcer Disease

This disease is caused by the gastric pathogen *Helicobacter pylori*, which produces CagA and VacA toxins [104,105,106]. These toxins have been identified as secreted by *H. pylori* EMN [107,108]—the latter undergo internalization with gastric epithelial cells by clathrin-mediated endocytosis [109]. The lipid microdomains of host cell membranes are involved in the uptake of these vesicles [110].

### 3.10. Gastric Cancer

Chronic *H. pylori* infection is linked to an enhanced carcinogenesis risk [111,112]. These bacteria secrete EMN that invade the gastric epithelium via BabA and SabA adhesins and have a stimulus on the development of gastric cancer. It has also been demonstrated that oncoproteins, CagA, and VacA are associated with *H. pylori* EMN [113]. Thuswise, EMN-mediated delivery of CagA and VacA toxins to the gastric epithelium is a mechanism of *H. pylori*-induced carcinogenesis [114].

### 3.11. Gastroenteritis

*Campylobacter jejuni* producing cytolethal distending toxin (CDT) is the most common causative agent of gastroenteritis [115]. EMN is involved in the delivery of *C. jejuni* proteins to host cells. Proteomic analysis of these EMN revealed 151 proteins, including CDT. *C. jejuni* EMN displays cytotoxic activity and induces an immune response of intestinal epithelial cells, which was not decreased by pretreatment of EMN with proteinase K or polymyxin B [116].

### 3.12. Hemolytic Uremic Syndrome (HUS)

This disease is triggered by enterohemorrhagic *Escherichia coli* (EHEC) mediated by the action of Shiga toxin (Stx) and hemolysin EHEC (EHEC-Hly) [117]. EHEC-Hly belongs to the RTX family, and part of the toxin exists as EMN-associated EHEC-Hly [57]. The latter was 80 times more stable than free EHEC-Hly. This EMN- associated toxin acts as the cell-binding protein and hemolysin and, thereafter, induces their apoptosis. EMN-associated toxin penetrates endothelial cells through the dynamin-dependent endocytosis, followed by subsequent activation of caspase-9 and caspase-3 that causes apoptotic cell death [118].

### 3.13. Cholera

This infectious disease is characterized by severe watery diarrhea that can lead to dehydration and death. The pathogen is the bacterium *Vibrio cholerae*, which produces cytolysin (VCC), a pore-forming toxin that that form transmembrane β-barrel channels via eukaryotic cells lysis [119,120]. It has been demonstrated that VCC is secreted in association with EMN, and their toxigenicity to erythrocytes and epithelial cells has been shown. EMN- related VCC initiates autophagy in target cells [121]. *V. cholerae* EMN’s inflammatory potential is mediated by the presence of active NTP1 and nucleotide-binding domains (NOD1 and NOD2) [122].

### 3.14. Cystitis and Pyelonephritis

These diseases are triggered by uropathogenic *Escherichia coli* (UPEC), generating a variety of virulence factors [123]. The set of toxins includes hemolysin and cytotoxic necrotizing factor 1 (CNF1), which allows UPEC to cause tissue damage and incapacitate immune effector cells [124]. CNF1 is closely associated with EMN. Vesicles containing CNF1 affect host cells, suggesting that CNF1 is transported by EMN in the active form. In addition, the histone-like protein H-NS plays a role in the suppression of CNF1 production and participates in the release of EMN in UPEC [125].

### 3.15. Listeriosis

This is an invasive infection caused by *Listeria monocytogenes*, which is one of the most dangerous pathogens [126]. Listeriolysin, a pore-forming toxin, is the main virulence factor [127]. It was established that *L. monocytogenes* can produce EMN. Proteomic analysis showed that EMN from wild-type *L. monocytogenes* contained 130 proteins. Proteins expression is regulated by promoter DNA binding factors, σ(B), for example, transporters (OpuCA and OpuCC) and cell division protein (FtsZ). σ(B) plays a key role in the production of EMN and protein components of EMN, that are involved in stress responses (heat, acid, and bile resistance) [128]. EMN prevents pore formation induced by LLO, which leads to the inhibition of autophagy but facilitates the lytic action on eukaryotic cells [129].

### 3.16. Periodontitis

The bacterium *Porphyromonas gingivalis* mainly causes an inflammatory disease in the periodontal cavity [130]. It has been shown that *P. gingivalis* EMN can attach and penetrating the gingival epithelial cells. Vesicle-associated DNA and RNA and EMN-mediated gene transfer from *P. gingivalis* have also been found [131]. After the adhesion of EMN to the host cells, vesicles are introduced through the lipid raft along the endocytic pathway into target cells, and are directed to endosomes and then to lysosomes. As a result, the B7-H1 receptor activates, which leads to the cell-mediated immune response [132]. EMN is also selectively enriched in virulence factors, for example, gingipains and LPS, allowing them to initiate inflammatory responses to *P. gingivalis* infection and epithelial tissue disruption [133]. Gingipains contained in EMN degrade the transferrin receptor and paxillin, which inhibit cell migration and lead to cell death [134]. The mechanism of periodontal pathogens’ action is mediated by TLRs [135]. *P. gingivalis* periodontopathic pathogen triggers also a range of other disorders: cardiovascular disease, cancer, diabetes, rheumatoid arthritis, and Alzheimer’s disease [136].

In addition to *P. gingivalis*, several other bacteria can also be important for periodontal disease. Among them is *Actinobacillus actinomycetemcomitans*, which secrete EMN containing virulence-related proteins such as OmpA, 28 kDa lipoprotein, and leukotoxin can lead up to host cell lysis. The specific leukotoxic activity of EMN was 4–5 times higher than that of bacterial outer membrane preparations, which suggests the enrichment of these vesicles with leukotoxin [137]. These results indicate that EMN is an effective weapon for bacteria survival in the oral cavity and the stimulation of periodontitis and many other diseases.

### 3.17. Meningitis

This is an acute inflammation of the brain and spinal cord meninges caused by *E. coli* K1. This bacterium secretes hemolysin and CNF1, a GTPase-activating bacterial toxin [138]. The latter promotes the invasion of *E. coli* K1 into the human brain of microvascular endothelial cells, representing the blood–brain barrier. EMN is involved in the delivery of CNF1 to host cells. YgfZ, a periplasmic protein, mediates the sorting of CNF1 into EMN. The EMN-CNF1 insertion into the brain results from the interaction with its laminin receptor. Accordingly, YgfZ promotes the invasion of CNF1 in the brain and vesicle involvement in triggering meningitis [139]. α-Hemolysin is a meningitis provocative virulence factor of the pathogenic *E. coli* (ExPEC). Internalization of EMN containing α-hemolysin by the red blood cells led to their lysis [140].

### 3.18. Anthrax

The cause of the disease is the Gram-positive bacterium *Bacillus anthracis*, which is a powerful biological warfare agent. *B. anthracis* secretes anthrax toxin, which consists of a protective antigen, an edema factor, and a lethal factor [141]. The protective antigen binds to the membranes of target cells and is cleaved by proteases into two fragments. The larger fragment serves as a receptor for edema and lethal factors and arranges their endocytosis [142]. Edema factor is a calmodulin-dependent adenylate cyclase. The use of calmodulin and ATP increases cAMP concentration. As a result, the neutrophil function is restrained. The lethal factor, entering the cell with the help of a protective antigen, causes its death [143]. Anthrax toxin was identified in EMN isolated from *B. anthracis*. mAbs of the protective antigen served to protect macrophages against EMN. The results show that EMN-associated secretion of *B. anthracis* toxin provides concentrated delivery of toxin components to target host cells [144].

### 3.19. Tularemia

The cause of the disease is the Gram-negative, facultative intracellular bacterium *Francisella tularensis* being the utmost contagious pathogenic bacterium that has been acknowledged as a potential biological warfare weapon. The bacterium sheds EMN in an unusual form of nanotubules that bear toxins and other virulence factors, suggesting their implication in pathogenesis. The EMN contains compounds (specifically OM proteins, LPS, and lipids) with immunomodulatory competence that allows an interaction with host PRRs and thus inducing the signaling process that led to enhanced output of chemokines, cytokines, and various inflammatory peptides [145].

### 3.20. Edvarsiellosis

Infection with *Edwardsiella tarda* causes sepsis and emphysematous putrefactive disease (edvarsiellosis) [146]. EMN secreted by these bacteria plays an important role in pathogenesis. The proteomic analysis revealed 74 proteins in the vesicles, including many virulence factors such as hemolysin, OmpA, porin, GAPDH, EseB, EseC, EseD, EvpC, EvpP, and lipoprotein. When EMN was injected into the organism, a significantly more intense induction of IL-1β, IL-6, TNFα, and IFNγ was observed compared to the levels observed upon injection of formalin-killed *E. tarda* [147].

### 3.21. Endocarditis

Inflammation of the heart endocardium is caused by bacteria, such as *Aggregatibacter actinomycetemcomitans*, *Haemophilus* spp., *Cardiobacterium hominis*, *Eikenella corrodens*, and *Kingella kingae* [148]. EMN secreted by these bacteria can deliver several proteins, including the toxin CDT, to the fibroblasts. EMN internalizes into the host cells, translocates into the endoplasmic reticulum, and carries antigens that induce NOD1- and NOD2-dependent NF-κB activation. Besides that, NOD1 has the function of a peptidoglycan sensor delivered by EMN to the host cells, which participates in the inflammatory responses caused by vesicles [61].

### 3.22. Atherosclerosis

Many pathogens that cause chronic infectious diseases, such as pneumonia, periodontitis, and others, make a significant contribution to the development of atherosclerosis [149]. Pathogen-originated DNA from bacteria, for example, *Porphyromonas gingivalis*, *Actinobacillus actinomycetemcomitans*, *Prevotella intermedia*, *Tannerella forsythia*, *Chlamydia pneumoniae*, *Helicobacter pylori*, *Mycoplasma pneumoniae*, *Treponema denticola*, *Staphylococus aureus*, *Prevotella intermedia*, and *Streptococcus mutans* has been detected in atherosclerotic plaques of patients with a high detection frequency [150]. *C. pneumonia* and *P. gingivalis* promote the expression of adhesion molecules, activate and bind TLR 4, and upregulate the expression of Vascular cell adhesion molecule-1, Intercellular adhesion molecule-1, and ox-LDL receptor [151]. *P. gingivalis* infection accelerates atherosclerosis development, which is related to a rise in the expression of TLR2 and TLR4 in atherosclerotic lesions [152]. Additionally, monocyte chemoattractant protein-1 is triggered by the variety of pathogens, such as *C. pneumoniae* and other periodontal organisms [153]. Experimental data demonstrate that Helicobacter pylori infection is involved in atherosclerosis development using EMN-based mechanisms. These nanostructures, carrying cytotoxin CagA, have been detected in blood circulation [154]. EMN of *H. pylori* administer the transport of virulence factors, such as CagA, to blood vessel epithelial cells that downregulates the expression of transcriptional factors PPARγ and LXRα, and thus promotes foam cell formation from macrophage, and accelerates atherosclerotic plaque growth [155]. These results shed light on the EMN’s role in the pathogenesis of infection-associated atherosclerosis.

## 4. EMN Application in Medicine: Current State and Future Directions

The knowledge on EMN accumulated to date provides an opportunity to develop approaches for a fairly wide range of their applications. Undoubtedly, these bacterial nanoparticles will be used in many areas of biotechnology and medicine [14,29,40]. For instance, EMNs are information carriers [156]. Thereby circulating EMN is an indicator of various diseases severity [157], and EMN-based diagnostic tools for bacterial infections [158]. Particularly, they can be used as biomarkers [159], drug delivery systems, tissue regeneration, anti-bacterial adhesion agents, and vaccine carriers [8,40,80,160].

EMN containing PAMPs are the main instrument of bacteria that initiate the host immune response. They can perform this in two different ways: either by prompting the manufacture of immune effectors or by stimulating the production of inflammatory mediators. During this process, leukocytes and other types of immune cells are recruited. PRRs, which are present at the leukocyte surface, come in contact with PAMPs, thereby initiating cell-signaling cascades and releasing various signaling molecules, such as cytokines, chemokines, and other mediators of inflammation, like reactive oxygen species. All of these host-derived molecules, as well as EMN themselves, can be detected in all the body biofluids and are superb biomarkers for the detection of infectious diseases [8,161].

Another important EMN application is the targeted delivery of drugs, proteins, lipids, oligonucleotides, RNA, and DNA to the desired organs and tissues [42,162], including antibiotics [163] and other therapeutic targets [14,164]. This implication represents a severe challenge in the field of therapy. EMN is usually extracted from the cultured cells via centrifugation under various conditions depending on the bacterial species and the EMN properties. The usual particle size is 40–200 nm. Essential impediments for drug-specific penetration are their rapid removal, difficulty in entering cells, toxicity, and immune barriers. Several approaches have been proposed, such as viral vector technologies, liposome carriers, peptide conjugation, and others. However, there are significant obstacles to the use of these methods, such as manufacture complicacy, inactivation, cost, toxicity, stability, and other issues [165]. A good alternative is EMN delivery of therapeutics [8,160]. Owing to the capacity of bacterial EMN to transport various moieties to host cells and other bacteria, they can be used to deliver drugs to the target organ, tissue, or cells [163].

The 21st century is characterized by the entry into the post-antibiotic era, which is explained by the total expansion of bacterial strains with multidrug resistance. Infections that could be treated by antibiotics can now leads to death. In this regard, there is an urgent need to discover new therapeutic strategies. Particularly, antibiotic targeted delivery is the most important. In this regard, there is an urgent need to develop more advanced approaches for guaranteed and safe antibiotic delivery to tissues and cells. EMN is an excellent alternative for implication in antibiotic therapy [40,163,166].

## 5. Vaccine Development Based on EMN

Interest in EMN as a vaccine carrier is growing as far as investigations uncover more of vesiculation molecular features in bacteria and the potential for EMN to be used to fight infectious diseases [15]. Last two decades, EMN began to be actively exploited to create vaccines against infectious diseases caused by pathogenic bacteria. Indeed, the EMN surface contains virulence factors and toxins, which are antigens in native conformation that have the properties of immunogenicity, natural adjuvants, and the ability to easily invade immune cells [167,168,169]. Currently, several EMN-based vaccines are developing against various infectious diseases (Table 1).

### 5.1. EMN Vaccines against Cancer

**Figure 2:**
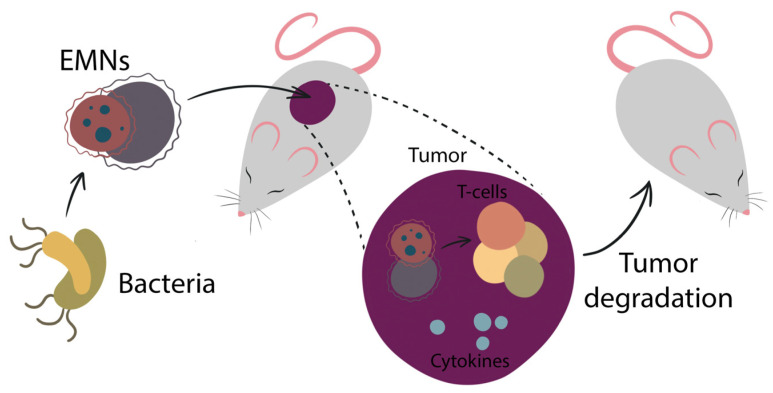
After isolation from bacteria, EMNs are modified to carry essential for tumor eradication components, such as chemotherapy agents. This leads to tumor degradation.

Recently, EMN role in inducing immunity not only against bacterial infections but also against cancer cells became interesting in the therapy scope. EMN, as carriers to deliver tumor antigens in cancer therapy, can bear heterologous antigens targeting tumor cells. EMNs have a broad potential to be applied in cancer management. First, bacteria-associated substances were reported to use against cancer in the 1890s, when Dr. William Coley administered weakened bacteria to cancer patients. The beneficial effect was observed, which was due to the immune system activation caused by bacteria immunostimulatory molecules. Unfortunately, the safety of such an approach appeared to be the main limitation. In this scope, the bacteria mimicking EMNs can be a safer option in comparison with live bacteria, as they are non-replicating. Moreover, EMNs can have a variety of immunostimulatory molecules, which enable the recognition and uptake of EMNs by immune cells, that leads to subsequent activation of the immune system. Additionally, the nano-scaled size of EMN particles allows them to accumulate within the tumor and thus stimulate local immunity via an increased permeation and retention (EPR) effect. All this makes EMNs an important candidate to be involved in the management of cancer. This approach is schematically represented in Figure 2.

EMN is an ideal instrument in this field of medicine, having good adapted structural and functional features, including their nano-scale dimension, natural adjuvant capacity, endowments to genetic and protein modifications, efficiency to present exogenous proteins, and availability of immune stimulators [189].

Currently, a lot of different bacterial vectors exist, that can be used for build-out EMN-based cancer vaccines [190]. Genetically engineered bacterial EMN is capable to target and kill tumor cells by transferring small interfering RNA (siRNA). An engineered mutant *E. coli* strain with decreased toxicity toward human cells was able to produce EMN bearing a human epidermal growth factor receptor 2 (HER2)-specific targeting ligand. The insertion of EMN loaded with siRNA caused targeted induction of significant tumor growth retardation. Thus, engineered EMN has a great capability as a drug-delivery facility to fight against cancers [191].

After invading a cancer tissue, these constructs induce innate and adaptive immunity and thus destroy tumor cells. To deliver target-oriented proteins or peptides with toxic properties to tumor cells, EMN must tie together to target cells by interactions between ligand and receptor.

This is realised through TLR binding. This is followed by the release of signaling molecules, cytokines, and chemokines, inactivation of CD4+ and CD8+ T cells, and extinguishment of regulatory T cells. EMN also promotes the IFN-γ emission and T cell-bound anti-tumor effects. An engineering construct was developed, where a programmed death 1 domain was built into the EMN surface. The engineered EMN-PD1 is associated with PD-L1 on the cancer cell surface, which results in the impairment of tumor growth. These data demonstrate the competence of bioengineered EMN to raise anti-tumor usefulness [192].

Thus, the EMN of *Salmonella Typhimurium* showed antitumor activity in human colorectal carcinoma and breast cancer [193]. Bacterial EMN-Cancer Cell hybrid membrane vesicles were successfully administered for immunotherapy together with photothermal therapy to improve the antitumor efficacy toward melanoma [194].

Owing to their ability to influence immunoregulation and ableness to deliver recombinant antigens to host cells, EMN can serve as an effective therapeutic vaccine against cancer cells through genetic engineering [189].

### 5.2. EMN Engineering

In the last decade, a growing number of works have appeared on the development of EMN with modified properties using a variety of molecular biology methods, and genetic and protein engineering. Because EMN is a promising facility for various substances delivery to recipient cells, engineering exploits the possibility to carry out the expression of recombinant proteins of interest in bacterial periplasmic space, which will be packaged in EMN. Several different approaches to load vesicles with the necessary therapeutic substances, such as saturation of the periplasmic space with the recombinant protein of interest or using DNA implantation into bacteria via electroporation [8,160,165]. One of the highly promising methods of engineering is the direct anchoring of recombinant proteins to the EMN surface [195,196,197]. The need for such an EMN-constructions development is also prompted to improve their capabilities, reduce the EMN toxicity, increase the immunogenicity and gain better productivity by parent bacteria for biotechnological and medical applications. Recent works have shown that genetically engineered EMN can overexpress antigens [163,187,198]. Thus, recombinant *N. meningitidis* EMN has been created, where *Borrelia burgdorferi* antigens OspA and OspC were expressed for obtaining an anti-Lyme disease vaccine [199]. Additionally, *E. coli* EMN was used as a platform for delivering *M. tuberculosis* antigens ESAT6, Ag85B, and Rv2660c to the targeted host. This engineered construct has been retrieved upon the tethering of recombinant antigens to the bacterial vesicle surface by the fusion to hemoglobin protease protein. Apart from that, a recombinant product was established based on hypervesiculating *Salmonella enterica* EMN with incorporated multiple heterologous antigens, namely, *M. tuberculosis* antigens and epitopes from *Chlamydia trachomatis* major outer membrane protein. Furthermore, it was demonstrated that *Salmonella* EMN transfer of recombinant Ag85B to immunocompetent cells conduce to the presentation of an epitope that is functionally recognized by T cells [173].

Engineered vaccines have been, in particular, developed to fight the coronavirus infectious disease, caused by SARS-CoV-2. It was the SARS-CoV-2 vaccine based on the EMN of *S. typhimurium* with incorporated spike RBD-domain from mammalian cells. After immunization, high titers of blood anti-RBD IgG were detected [200]. EMN-emitting bacteria can undergo genetic handling or metabolic engineering to produce surface-modified extracellular vesicles for vaccine technology and targeted drug delivery [159].

Genetic modifications are now the most promising strategy for making EMNs suitable for use. However, the distribution of antigens and lipids on the bacterial membrane is unbalanced, which makes the engineering process even more complicated. Further studies in the fields of vesiculation mechanisms would provide the possibility of creating engineered MVs enriched in ideal components for the application.

## 6. Conclusions

The data presented in the literature show that EMN secretion is an evolutionarily fixed, universal process immanent in all bacterial species. The main EMN role emitted by Gram-negative, Gram-positive, and archaebacteria is the provision of communication with other cells of prokaryotes and eukaryotes. EMN production is essential for the survival of bacteria and their adaptation to environmental conditions. Extracellular vesicles allow pathogenic bacteria to deliver virulence factors to distant targets in a concentrated and protected form.

The discovery and exploration of bacterial EMN have allowed medicine in the field of infectious diseases to have passed to an outright new level. A growing number of studies have established that EMN represents a powerful tool for impairment host cells. It gave a new insight into the pathogenesis of infectious diseases. Now the complex secretion mechanisms of pathogenic bacteria’s toxins and virulence factors within EMN, their targeted delivery to host cells, adhesion, internalization, and intracellular trafficking have begun to be discovered. The high concentration of virulence factors, the ability to accurately target them, the easiness of penetration into host cells, and natural adjuvant capabilities have allowed the EMN to be applied as highly effective vaccines. To increase the effectiveness of extracellular vesicles as vaccine carriers and therapeutics delivery vehicles, as well as to reduce the toxicity of some components of EMN, methods of genetic and protein engineering are exploited. Studies have shown that EMNs also have ample opportunities for their use in other areas of medicine. In particular, they can be used to diagnose infectious diseases, for delivering antibiotics, and other drugs to the desired body tissues. Undoubtedly, further in-depth EMN study and the molecular mechanisms underlying all physiological processes associated with these nanovesicles will lead to new advances in medicine.

EMNs are a promising candidate for vaccine development, but, unfortunately, they exhibit several limitations. Among them are the high reactogenicity of pathogen-associated molecular patterns, low expression levels of relevant protective antigens, strain variation resulting in many subtypes of specific antigens, thus lower coverage, immuno-dominant antigens that misdirect the immune response, and molecules, which are immunosuppressive or otherwise interfere with a protective immune response. Thus, genetic engineering methods can help to avoid, at least in part, these limitations. Potential approaches can be modifications, removal, and addition of EMN proteins and other components.

## Figures and Tables

**Table 1:** Vaccine candidates developing on the basis of EMN against diverse pathogenic bacteria.

Disease	Infectious Agent	Approach	References
Pneumonia	*Acinetobacter* *baumannii*, *Bordetella pertussis*, *Streptococcus* *pneumoniae*, *Klebsiella pneumonia*	intramuscular, intraperitoneal or the intranasal administration of *A. baumannii* EMNs, including multidrug-resistant strains; intranasal administration of *B. pertussis* EMNs; intranasal administration of native *S. pneumoniae* MVs; intraperitoneal administration of native *K. pneumoniae*-secreted EMNs	[83]
Meningitis	*Neisseria meningitides*	the complex formed from the adjuvant lipopolysaccharide (LPS) and bacterial surface antigen allows EMNs to produce an adaptive immune response	[170,171]
Periodontitis	*Porphyromonas* *gingivalis*	intranasal immunization with a combination of *P. gingivalis* EMNs and a TLR3 agonist, Poly(I:C)	[172]
Tuberculosis	*Mycobacterium* *tuberculosis*	Immunization with an Ag85B-ESAT6-Rv2660c fusion protein stimulated stronger immune responses and better protection against *M. tuberculosis*	[173]
Cystic fibrosis	*Pseudomonas* *aeruginosa*	Immunization with *Pseudomonas aeruginosa* EMNs was efficient to launch a complex humoral and cellular immune response in mice	[174]
Whooping cough	*Bordetella parapertussis* *Bordetella pertussis*	intranasal administration of *B. pertussis* EMNs	[175,176]
Sepsis (Melioidosis)	*Burkholderia* *pseudomallei*	multivalent EMN vaccine derived from *B. pseudomallei* strain 1026b	[177]
Plague	*Yersinia pestis*	Immunization with EV of engineered strain of *Y. pestis* producing a detoxified 1-dephosphorylated hexa-acylated version instead of highly inflammatory bisphosphoryl hexa-acylated lipid A (monophosphoryl lipid A, MPLA)	[178]
Gonorrhea	*Neisseria gonorrhoeae*	Intravaginal administration of EMN preparation with microencapsulated IL-12 as an adjuvant appeared to accelerate gonococcal clearance and to induce *N.gonorrhoeae*-specific antibodies to BALB/C mice	[179]
Peptic ulcer disease	*Helicobacter pylori*	Intragastrical immunization with the *H. pylori* EMNs plus CT as an adjuvant, or a WCV supplemented with CT	[180]
Hemolitic uremic syndrome	*E. coli* (STEC)	chemical-inactivated EMN (EMNi) obtained from a virulent STEC strain (O157:H7 serotype)	[181]
Gastroenteritis	*Campylobacter jejuni*	In ovo vaccination with wtEMNs, 639-EMNs, and 1405-EMNs of chicks	[182]
Cholera	*Vibrio cholera*	intranasal, intragastric, or intraperitoneal administration of EMNs derived from *V. cholerae*	[183]
Tularemia	*Francisella* *novicida*	EMNs were shown to induce both cell mediated and humoral immunity	[184]
Cystitis and pyelonephritis	*Escherichia coli* (UPEC)	OMPs purified from *E. coli* CFT073	[185]
Pertussis	*Bordetella pertussis*	EMNs derived from *B. pertussis*	[186]
Shigellosis	*Shigella flexneri*	EMNs derived from *S. flexneri*	[187]
Brucellosis	*Brucella melitensis*	Pluronic P85 enhances the efficacy of *B. melitensis* EMNs	[188]
Edwardsiellosis	*Edwardsiella tarda*	EMNs naturally released from *E. tarda* injected into olive flounders	[147]

## Data Availability

Not applicable.
